# Learning how to learn

**DOI:** 10.2349/biij.4.1.e10

**Published:** 2008-01-01

**Authors:** BJJ Abdullah

**Affiliations:** Department of Biomedical Imaging, Faculty of Medicine, University of Malaya, Kuala Lumpur, Malaysia

In proportion to our body mass, our brain is three times as large as that of our nearest relatives. This huge organ is dangerous and painful to give birth to, expensive to build and, in a resting human, uses about 20 percent of the body’s energy even though it is just 2 percent of the body's weight. There must be some reason for this evolutionary expense [1].

Recently, I lost one of my aunts. She was a self-made woman who came from very humble beginnings. She grew up in a village and did not have much in terms of any formal education. Despite these challenges, she managed to make a living by providing tasty food to the multitude of city workers during breakfast and lunch in a small stall in the city. She raised a family and had several grandchildren. When I think of her and compare her with my late father, I see a major difference. Even though my dad was also born in a little rural village, left his family when he was a child, came to another country, had minimal formal education, and did a multitude of different jobs, he learnt to read and write in several languages, he managed to send all his children for tertiary education and was knowledgeable about what was happening in the world. In addition, he was a respected community leader. Both were successes in their own different ways considering the limitations imposed on them as a result of their backgrounds.

Why is it that some are able to rise above their backgrounds while others are not able to do so? Are the differences genetic or environmental or a combination of both, the perpetual nature versus nurture question? Why is it some are never happy with where they are, always striving to do better? How can we have better means of communicating and learning? Why is it learning how to learn has never been an important part of our curriculum? Do we know that we have to unlearn what is no longer relevant so that we can learn new things? What measures can we take to reduce our stress levels? How much more each one of us could achieve if we truly knew the extent of our capabilities? Can we “regrow” bits of our brain that we lost? Can we improve our task performance just by thinking about it? How much better the working environment could be if we let people know their potential? What more can be done to push and drive people to excel, to discover themselves? Are there better ways of organising thoughts, have better retention and recall, and achieve higher creativity? How can one become more creative? Is it all about the mind, the way we think and act?

The current dilemma we face regarding the mind has been partly due to the metaphysical divide propounded by Rene Descartes, the great French mathematician and philosopher of the 17th Century. Even those of uswho come from the eastern traditions where the mind and body are seen as one have come to accept this duality to some degree from the western-based educational system that we have been through. Today, scientists, neurologists, psychologists and those dabbling in the brain recognise that the body and mind are not different but that they are just an invisible continuum. The mental and physical well-being of an individual is intimately intertwined since they share the same nervous, circulatory, endocrine and immune systems. What happens in the brain e.g. stress is directly transmitted to the adrenals, which secrete more cortisol and affect the entire body. An unhealthy body can lead to an unhealthy mind, and an illness of the mind can trigger or worsen diseases in the body. Fixing a problem in one place, moreover, can often help the other. Heart disease is one of many illnesses that worsens with depression. People with such afflictions as cancer, diabetes, epilepsy and osteoporosis all appear to run a higher risk of disability or premature death when they are clinically depressed. Depression really is a systemic disorder.

Our understanding of how the human mind (and maybe brain) functions has expanded tremendously over the last two decades. This has been, to a large extent, due to developments in medicine especially in imaging. With the advent of functional imaging with MRI, PET/CT as well as SPECT, the field of functional neuroimaging has exploded. How will this change what radiologists/images have been doing all this while. The change from looking at disease entities to using imaging to better understand the functioning of the brain, in current paradigms, is something that has not been emphasized. Unlike other areas of the body where imaging has not had much role in looking at normal physiological processes, the brain has been different. It has been an area of interest for neurologists, neurobiologists, neuroanatomist, psychologists, psychiatrists, and others using EEG, EMG, MEG, etc. to better understand the functioning of the brain. With the extensive knowledge the images have in this area, they can certainly add a lot more to the overall discussion and contribute towards a better understanding of the intricate functioning of the brain; its role in addiction, drug discovery, forensic diagnosis, in memory creation, learning, etc. However, this raises questions as to who has right to look into our heads when the times comes; the government, your employer, your family or your spouse? Does it then give the companies the right to sell you things you do not really want by seeing how you respond to different forms of advertising?

What has been even more troubling is our lack of emphasis on how all this new knowledge can be and should be spread amongst our trainees and staff who would be better able to learn and unlearn, to be more creative, to be able to better handle stress, to be life-long learners and also to understand how we could deliver better services for our patients and community. Both teachers and learners are not exposed to this crucial area of competency but have focused too much on acquiring professional and task-specific knowledge. Why is that so? Probably because we have forgotten the importance of understanding the functioning of the mind, too much to learn about one’s own area of expertise that there is not enough time for anything else, we were never taught about optimising learning and teaching the most updated knowledge, the constant discussion of the rights and wrongs of a particular study, familiarity, force of habit and desire to keep things the way they are, it is too much work to change things around when we are already doing fine!

We, as professionals, are guilty of concentrating on our core competencies and staying in touch with the rapid advances so much so we have forgotten that new information in the understanding of the mind will drastically change the way we teach, what we teach and how to keep learning by unlearning previous concepts and theories. Our teaching material is from the dark ages, in a sense, with emphasis on rote learningand didactic lectures, which are almost entirely in textual form, involves minimal interaction and are static. Newer ways of e-learning with the use of interactive, computer-based, dynamic and learner centric tools based on more current understanding of learning are the future of learning [2].

**Figure 1:**
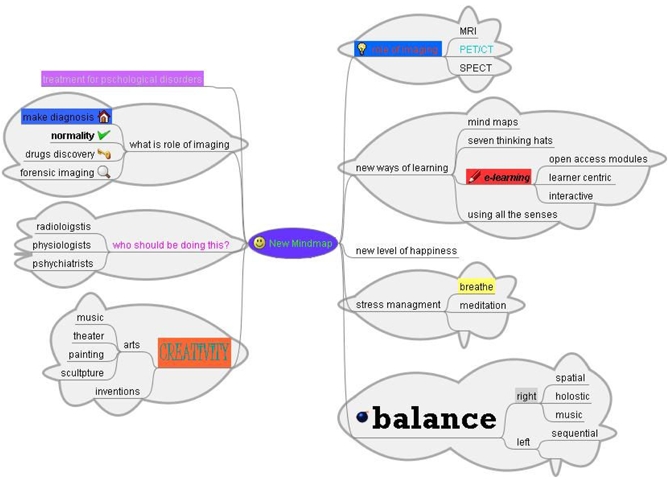
Mind map of learning to learn

What about mind maps (Figure 1)? Contrary to long-held beliefs of the brain being unchanging and that certain psychiatric conditions are hard-wired, it is now being accepted that the adult brain retains impressive powers of "neuroplasticity", which is the ability to change its structure and function in response to experience. This has been shown in those who experience phantom limbs over their faces or groin. Studies have shown practicing a move or action repeatedly in your brain over time can cause areas in the brain, which cover those movements, to expand. The effect is better than if you had done all this physically. This mental rehearsal of actions has been used by top athletes for a long time. They go through every step of their task from start to finish while visualising their bodies at every step. Should we be practicing our barium enemas, radium seed implants for brachytherapy or procedures for calibration of a CT scanner over and over in our heads so that the actual procedure would be easy? I have been doing this after learning about it from athletics. Constraint-induced movement therapy, a new form of therapy has been proposed to help stroke victims recover function by developing new areas (in their brain?).

Learning how to handle stress in our increasingly complicated and rapidly changing world is also important. When one thing changes, all the other related areas change to some degree and finding the new balance may not be all that easy. The mind has a desire to make a decision (hopefully right) quickly and this period of uncertainty is probably the reason why change is so difficult and traumatic. When too many rules change, when what used to work does not work anymore, your ability to reason takes a hit and you may get into a state of helplessness.

Can we learn to be more creative or is it god given? Contrary to what most believe that creativity is a thing or a state of mind that occurs suddenly or is even a gift, creativity is truly a process. It is a process that can be observed, analyzed, understood and even replicated, taught and managed [3]. When you're creative, your brain is using the same mental building blocks you use every day. Creativity does not happen in one brilliant flash but in a chain reaction of many thought processes ruminating on a problem. The creative process is basically the same: generating ideas, evaluating and executing them, with many mistakes over time. Collaborating with others is also key! When we take time off from working on a problem, we change our context, which can activate different areas of our brain where the solution may be. If we are lucky, in that new and different and totally unrelated context, we may hear or see something that relates distantly to our problem that we had temporarily put aside, and the solution miraculously appears. That is why the three Bs - for the bathtub, the bed and the bus are places where ideas have famously and suddenly emerged [4]. Did you know that? Did we even want to find out about it? Probably everything we know about it is from the newspapers or television. And why not? Most often we probably did not think it was important enough. How much more each of us could contribute if we could be 10% more creative? How much more could be achieved if we could help the people we live and work with, learn it?

The ultimate question that needs to be asked, knowing what we know about the mind thus far, is can we learn to be happy? The doom and gloom crowd would say without a doubt it is absurd. Eastern philosophy has always said that we can and that happiness is a full-time job. It is a state of mind. It is the ability to keep things in proportion by having a sense of humour. It can be practiced and mastered by leading purposeful lives for causes bigger than oneself and immersing yourself completely within your life. Scientific research is now understanding and agreeing with them [5]. Learn how to be appreciative of all that you have and share your love. Studies show that when people engage in appreciative activity, they are using more neocortical and prefrontal, i.e. higher-level, brain functions.

What we do in our working/professional/student lives is important since we are all contributing to our society and community in different ways but we often forget that work is actually another means of getting us to where we wish to go. Work provides learning opportunities for us to do better, to make a difference, to push our abilities even further, to make our workplace more enjoyable, to learn to love a little more, to live a lot better, and to give without expecting. If we miss these opportunities at work then we have not really learnt while we are “living” and working.

Be careful what you think because you can become it!
